# Morphological and structural alterations in skeletal muscle of STZ-induced diabetic rats revealed by light and electron microscopy

**DOI:** 10.5455/javar.2026.m1011

**Published:** 2026-03-05

**Authors:** Hanan Kumar Gopalan, Yanti Rosli, Noman D. Salih, Nur Afrina Muhamad Hendri, Wirda Indah Farouk, Nur Syahrunnizar, Mohd Shazwan Shazdee Wahab, Jyh Chyang Pang, Norzein Ab Rahim, Hiang Lian Hing

**Affiliations:** 1 Department of Clinical Laboratory Sciences, Institute of Medical Science Technology (MESTECH), Universiti Kuala Lumpur, Kajang 43000, Selangor Darul Ehsan, Malaysia; 2 Biomedical Science Programme, Centre for Toxicology & Health Risk Studies (CORE), Faculty of Health Science, Universiti Kebangsaan Malaysia, Kuala Lumpur, Malaysia; 3 Fallujah Teaching Hospital, Immunology and Serology Unit, Fallujah, Anbar, Iraq; 4 Electron Microscopy Unit, Institute for Medical Research, Ministry of Health, Shah Alam 40170, Selangor Darul Ehsan, Malaysia; 5 Department of Diagnostic Laboratory Service, Universiti Kebangsaan Malaysia Medical Centre, Kuala Lumpur, Malaysia; 6 Division of Diabetology, Endocrinology and Nephrology, Shiga University of Medical Science, Seta, Otsu, Shiga, Japan; 7 First City University College, Petaling Jaya 47800, Selangor Darul Ehsan, Malaysia

**Keywords:** Scanning electron microscopy, transmission electron microscopy, myofiber, type 2 diabetes, hyperglycemia

## Abstract

**Objectives:** This study investigates morphological and ultrastructural changes in skeletal muscles of diabetic rats induced by streptozotocin (STZ) using light microscopy, field-emission scanning electron microscopy (FESEM), and transmission electron microscopy (TEM). Together, these imaging methods allow for detailed visualization of tissue-level and subcellular changes in the gastrocnemius muscle, offering an integrated view of diabetes-related pathology.

**Materials and Methods:** Diabetes was induced in male Sprague-Dawley rats through a single intraperitoneal injection of STZ (at 65 mg/kg). After 30 days of diabetes maintenance, the rats were euthanized, and gastrocnemius muscle samples were harvested for Hematoxylin and Eosin (H&E), Masson’s Trichrome (MT), and histochemical staining. Morphological features were examined using FESEM, and ultrastructural organization was analyzed using TEM.

**Results:** Our findings confirm that hyperglycemia severely damages skeletal muscle in STZ-induced diabetic rats, characterized by extensive myofiber injury, atrophy, and mitochondrial disruptions. Ultrastructural analysis revealed extensive architectural disruptions and clear deviations from normal morphology.

**Conclusions:** Our study provides evidence of profound structural and cellular alterations in diabetic skeletal muscles and offers insights for developing strategies to mitigate pathological changes, preserve muscle integrity, and support functional recovery.

## 1. Introduction

The global prevalence of diabetes mellitus was 463 million individuals in 2019, and is expected to reach 700 million by 2045 [1]. Hyperglycemia, or an elevated blood sugar level, is commonly observed among individuals with this disease, in which insulin dysfunction occurs [2]. Diabetes mellitus can occur in two main types: type 1 diabetes mellitus (T1DM) and type 2 diabetes mellitus (T2DM). T1DM is an autoimmune condition in which pancreatic β-cells are destroyed, leading to decreased insulin production [3]. It accounts for less than 10% of all cases of diabetes mellitus [4]. It may manifest itself from early childhood, leading to serious complications of nephropathy, neuropathy, and retinopathy [4]. T2DM, which accounts for approximately 90% of all cases, occurs when the body becomes less sensitive to insulin – a condition known as insulin resistance [5]. This condition is caused by persistently high blood glucose levels. In the long term, it affects other organs, including the kidneys, eyes, and even skeletal muscle [6].

There are three major types of muscle in the body: skeletal, cardiac, and smooth. The musculoskeletal system relies heavily on skeletal muscle, which maintains posture, breathing, and homeostasis [7]. It also accounts for more than 80% of glucose uptake and is necessary for glucose clearance [2]. The remarkable regenerative potential of skeletal muscle is primarily due to a population of mature myogenic cells, known as satellite cells, which possess self-renewal capacity and can differentiate into new, multinucleated myofibers to repair and regenerate following injury. Nonetheless, elevated glucose levels can impair regenerative potential by reducing satellite cell proliferation [8]. Therefore, to achieve successful muscle regeneration, the muscle’s metabolic homeostasis must be preserved. However, disorders of glucose metabolism, such as diabetes mellitus, may significantly influence muscle function [9].

Diabetic myopathy refers to the slowly progressive weakness of muscles resulting from disturbed metabolism or myopathy associated with diabetes. Skeletal muscle becomes dysfunctional in its structure, metabolism, and overall function, all of which are characteristic features of this debilitating disease [10, 11]. In T1DM patients, myofibrillar size, glycogen levels, and mitochondrial activity have all been found to change [12]. By differentiating into multiple cell types, including fibroblasts, adipocytes, and osteocytes, in response to environmental cues, fibro-adipogenic precursors (FAPs) regulate extracellular matrix (ECM) remodeling and fibrosis in a mouse model [13, 14]. The increased collagen content in T2DM patients was correlated with an increase in FAPs proliferation [15].

Although numerous literature reviews have examined the effects of diabetes mellitus on skeletal muscle, no subsequent studies have investigated structural changes in skeletal muscle associated with T2DM using advanced electron microscopy techniques, such as field-emission scanning electron microscopy (FESEM) or transmission electron microscopy (TEM). We aim to address this gap by studying these ultrastructural alterations using these two cutting-edge techniques.

## 2. Materials and Methods

### 2.1. Ethical approval

The use of animals in this project has been approved by the Universiti Kuala Lumpur (UNIKL), MESTECH (AEC/MESTECH-UNIKL/2019/2021/JULY 2019-July 2021).

### 2.2. Selection of animals and animal care

Twenty adult male Sprague-Dawley rats (250–300 gm) were randomly assigned to two groups; which were normoglycemic (*n* = 10) and streptozotocin (STZ)-induced (*n* = 10). The animals were acclimated for 7 days and were kept under standard laboratory conditions (a 12-h dark-light cycle). They were allowed free access to food and water during the entire experimental period. Five rats were used for light microscopy, and the remaining five for electron microscopy (FESEM and TEM). This allocation ensured sufficient replicates and sample size for both groups.

### 2.3. Induction of type 2 diabetes

Type 2 diabetes was induced in the rats after fasting for 12 h through an intraperitoneal injection of streptozotocin (Santa Cruz Biotechnology, Inc., Santa Cruz, CA, USA), dissolved in normal saline at a dose of 65 mg/kg. The low-dose STZ model was selected to induce moderate and sustained hyperglycemia, which closely resembles the development of T2DM rather than the extensive β-cell destruction observed in high-dose STZ models used for T1DM. The present method allows the development of a non-insulin-dependent diabetes mellitus (NIDDM) state, reflecting insulin resistance and partial β-cell dysfunction, as characteristic features of T2DM, using the protocol described by Rehman et al. [16].

Diabetes was confirmed by an increased level of blood glucose 14 days after injection, along with clinical signs including polydipsia and polyphagia. Rats were included in the study if they had sustained NIDDM, defined as a fasting blood glucose level greater than 170 mg/dl for at least 1 week. All rats in the diabetic group developed NIDDM one week later. All efforts were made to minimize suffering, and the ethical guidelines for animal experimentation were strictly followed. All rats were monitored for any physiological or metabolic modifications for one month. Body weight and diabetes mellitus symptoms were frequently monitored in the diabetic group. The normoglycemic rats served as controls and provided a reference for testing the effects of STZ-induced diabetes mellitus on skeletal muscle.

### 2.4. Histological observation

At the end of the 30 days, the rats were euthanized, and gastrocnemius muscle samples were excised and fixed in 10% neutral buffered formalin (SYSTERM^®^, Malaysia) for 24 h. Muscle samples were then cut into 5 mm thick segments and dehydrated through a series of graded ethanol solutions (70%, 80%, 95%, and 100%) followed by clearing in xylene (Thermo Fisher Scientific^®^, USA). The processed tissue was then embedded in paraffin wax (Leica Paraplast^®^, Germany). Paraffin sections were sliced into 4 µm sections using a microtome and mounted onto glass slides. The slides were then deparaffinized in xylene and subjected to staining with Hematoxylin and Eosin (H&E) (Leica Biosystems^®^). Slides were then serially dehydrated through an ascending series of absolute alcohol, followed by mounting with DPX and cover-slipping to visualize cellular and tissue structures.

### 2.5. Masson trichrome (MT) staining

Tissue sections of 4 μm thickness were deparaffinized in xylene (Thermo Fisher Scientific^®^, USA) and rehydrated through a graded series of descending alcohol concentrations. The slides were stained with a Masson›s trichrome (MT) staining kit from Bio–Optica^®^ (Milan, Italy) according to the manufacturer›s protocol. The stained slides were dehydrated serially through an ascending series of absolute alcohol. The slides were then mounted in DPX, cover-slipped, and observed under a microscope.

### 2.6. Histochemistry staining

Muscle specimens were snap frozen in isopentane (Sigma-Aldrich, USA) and cooled in liquid Nitrogen. The snap-frozen muscles were then embedded in optimal cutting temperature (OCT) compound (Sakura Finetek^®^, USA) and stored at –80°C prior to cryosectioning. Cryosections of 10-μm-thick transverse slices were sliced using a cryostat. These sections were mounted on glass slides for histochemical staining utilizing staining kits from Bio-Optica^®^ (Milan, Italy). Cytochrome C oxidase (COX) activity was detected using Cytochrome C and 3,3’-diaminobenzidine (DAB), which produced brown precipitates that marked regions with high oxidative activity. Succinate dehydrogenase (SDH) activity was assessed using succinate, resulting in the formation of blue-purple formazan deposits in regions of high mitochondrial activity. All staining procedures were performed according to the manufacturer’s protocols.

### 2.7. Morphological evaluation (FESEM)

Muscle specimens were fixed in 2.5% glutaraldehyde (Electron Microscopy Sciences™, PA, USA) in 0.1 M phosphate buffer for 24 h at room temperature. This was followed by post-fixation in 1% osmium tetroxide (Electron Microscopy Sciences™, PA, USA) for 1 h to enhance electron density and contrast. Dehydration was achieved using a graded ethanol series 50%, 70%, 90%, 100% (SYSTERM, Malaysia^®^) to preserve tissue morphology. Critical point drying was carried out using hexamethyldisilazane (Sigma-Aldrich^®^, USA) to complete the dehydration process. The dried specimens were then mounted on aluminum stubs with conductive adhesive (TED PELLA^®^, USA) and coated with a thin, uniform gold layer (~38 nm) using a sputter coater. High-resolution ultrastructural analysis was performed with FESEM (FEI Tecnai^®^, USA) at the Electron Microscopy Unit, Institute for Medical Research, Ministry of Health Malaysia.

### 2.8. Ultrastructural TEM evaluation

Rat gastrocnemius muscle samples were prepared for detailed ultrastructural analysis using TEM. Initially, the samples were washed three times with 0.1 M phosphate-buffered saline to remove debris and then fixed overnight in 3% glutaraldehyde (Electron Microscopy Sciences™, USA). The samples were then embedded in 3% agar and further fixed in 3% glutaraldehyde in 0.1 M PBS. Post-fixation was carried out using 1% osmium tetroxide (Electron Microscopy Sciences™, PA, USA) in 0.2 M PBS, followed by rinsing and staining with 3% uranyl acetate (TED PELLA^®^, USA). Dehydration was performed through a graded series of ethanol (SYSTERM^®^, Malaysia) and acetone (Chemiz, Malaysia), after which the samples were infiltrated with resin and embedded. Semi-thin sections (1 μm) were cut with a Leica MZ 6 ultra-microtome (Leica Biosystems™, USA), stained with toluidine blue, and examined using a Leica DM1000 optical microscope (Leica, USA) to identify areas of interest. Ultrathin sections (70–90 nm) were then prepared with an Ultra 35° diamond knife (DIATOME^®^, USA), collected on copper grids (TED PELLA^®^, USA), and stained with uranyl acetate and lead citrate. The sections were examined with a transmission electron microscope (FEI Tecnai^®^, USA) operating at 120 kV, at the Electron Microscopy Unit, Institute for Medical Research, Ministry of Health Malaysia.

## 3. Results

**Figure 1.**
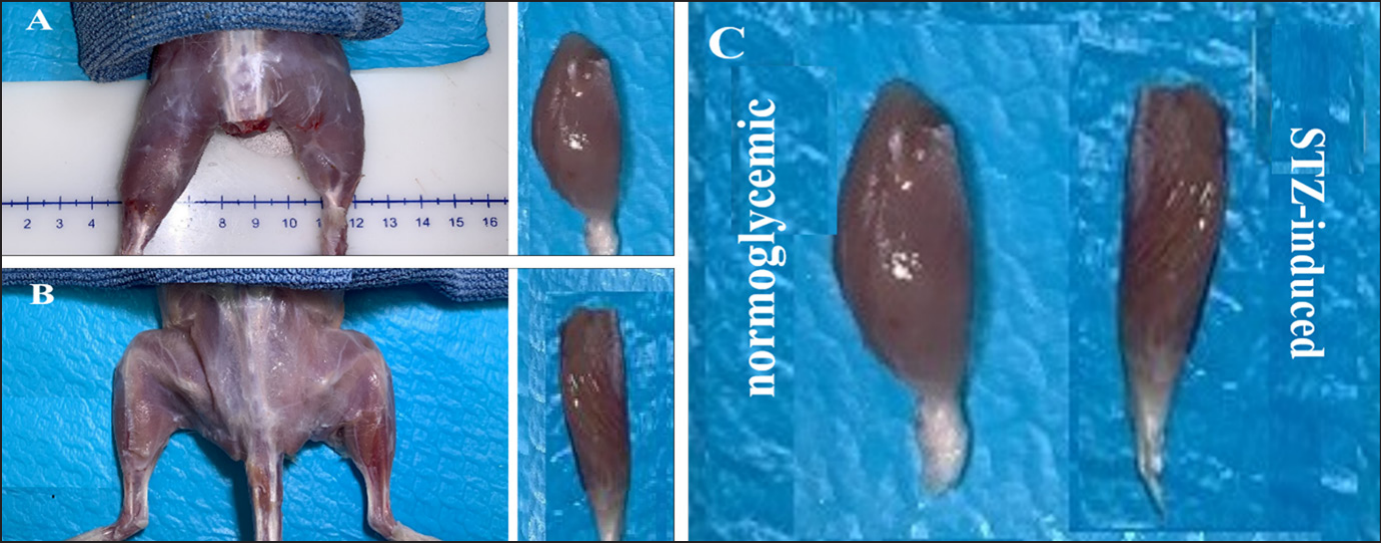
Gross anatomical photographs of a normoglycemic control rat (A), STZ-induced diabetic rat (B), and side-by-side comparison of the gastrocnemius muscle between normoglycemic and STZ-induced groups (C).

Visual assessment of the gastrocnemius muscle revealed clear morphological differences between normoglycemic (Figure 1A) and STZ-induced groups (Figure 1B). Normoglycemic rats displayed well-preserved muscle with uniform color and apparent bulk, whereas STZ-induced diabetic rats exhibited atrophy characterized by visibly reduced size and paler coloration (Figure 1C). These observations indicate that T2DM induces pronounced gross structural alterations in skeletal muscle, reflecting the impact of hyperglycemia on muscle integrity.

### 3.1. Architectural evaluation of gastrocnemius muscles

**Figure 2.**
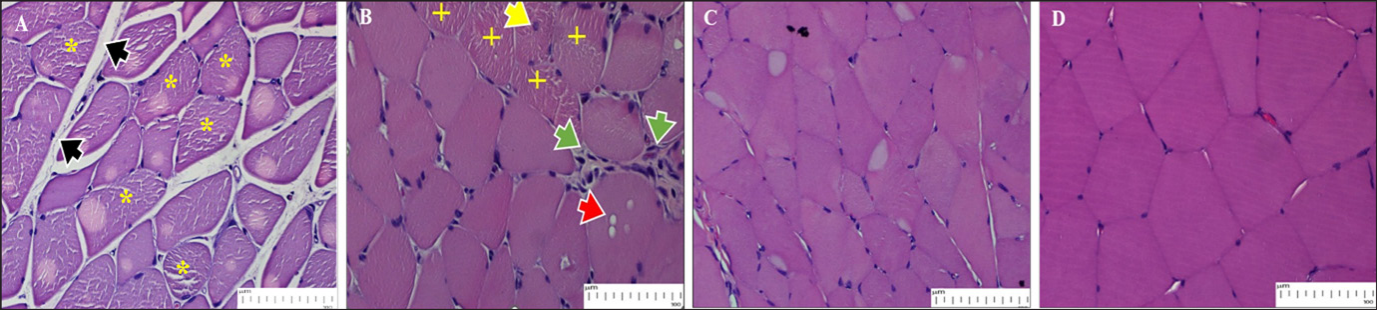
H&E staining of gastrocnemius muscle histological sections from STZ-induced diabetic rats (A, B, and C) and a normoglycemic rat (D). The muscle architecture of STZ-induced diabetic rats was significantly altered, with the fibers becoming disrupted and disordered. (A) Areas of myofiber degeneration or necrosis were indicated by widened intracellular spaces (black arrowhead) and focal hypereosinophilic regions (*). (B) Centralized nuclei (yellow arrowhead) are visible, suggesting a continued regenerative response to extensive damage of muscle fibers. Fiber atrophy (+) and cytoplasmic vacuolation, which also indicated myofiber degeneration, were observed. Inflammatory cells also appeared to migrate into the area (green arrowhead), suggesting that the muscle was damaged due to inflammation. (C) The size of the fiber appeared to be smaller than (D), demonstrating fiber atrophy. (D) The muscles of normoglycemic rats appeared normal.

H&E staining of the gastrocnemius muscles showed distinct histological features and structural changes. In STZ-induced diabetic rats (Figures 2A, B, C), muscle tissues presented evident architectural damage characterized by intracellular disorganization of fibers and heterogeneity in the shape and size of fibers, which is indicative of atrophy. The focal regions of hypereosinophilia reflected degenerating myofibers and necrotic fibers, while the presence of central nuclei indicated regenerative activity within the damaged fibers.

Furthermore, vacuolation and infiltration of inflammatory cells were also observed, indicating that the inflammation contributed to the severity of muscle damage. In comparison, the gastrocnemius muscle from normoglycemic rats (Figure 2D) showed normal tissue structure and fiber orientation.

### 3.2. Histochemical analysis

**Figure 3.**
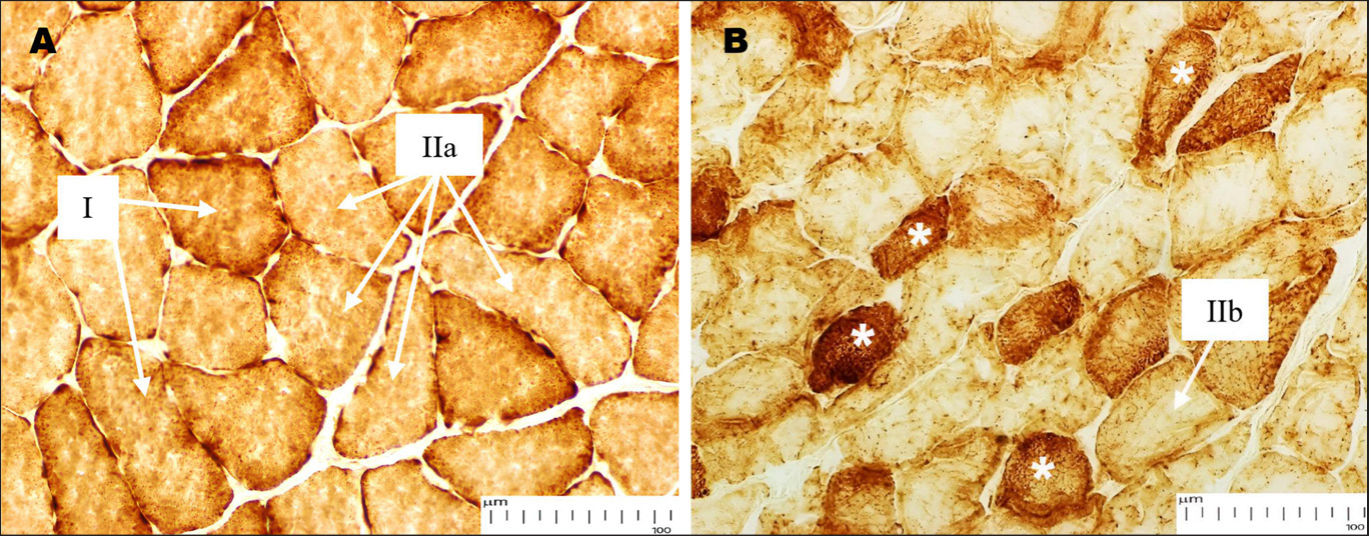
COX staining of the gastrocnemius muscle in normoglycemic (A) and STZ-induced diabetic rats (B). (A) In normal rats, strongly stained fibers also exhibited high COX staining (i.e., a high mitochondrial density and oxidative capacity), predominantly in slow oxidative fibers (Type I). The pronounced staining was indicative of an oxidative metabolism. On the other hand, moderate COX staining indicates reduced mitochondrial activity, mainly limited to Type IIa (fast glycolytic/oxidative) fibers that depend on a combination of oxidative and glycolytic metabolism. (B) STZ-diabetic groups showed moderate COX staining intensity in most of the muscle fibers, indicating a low mitochondrial density that is mainly present in type IIb (fast glycolytic) fibers. This is in accordance with the development of diabetes, and this staining pattern indicates their dependence on anaerobic glycolysis due to a change in muscle fiber composition. The heavy staining in some abnormal, irregular-shaped fibers (*) appears to be a compensatory reaction to the mitochondrial disorder.

Histochemical study of the gastrocnemius muscle reveals apparent pathological differences in normal and STZ-induced diabetic rats. Muscle staining of COX in the normoglycemic (Figure 3A) exhibited dark and light fibers. The presence of these two intensities is in accordance with the presumed distribution of oxidative (Type I) and fast-twitch fibers (Type IIa). This implies that the muscle has a high oxidative capacity. In the diabetic rats (Figure 3B), most fibers were pale, indicating a transition from an oxidative fiber type to a glycolytic type (Type IIb). This shift is concomitant with a decrease in oxidative capacity, a feature of metabolic disease, including diabetes.

**Figure 4.**
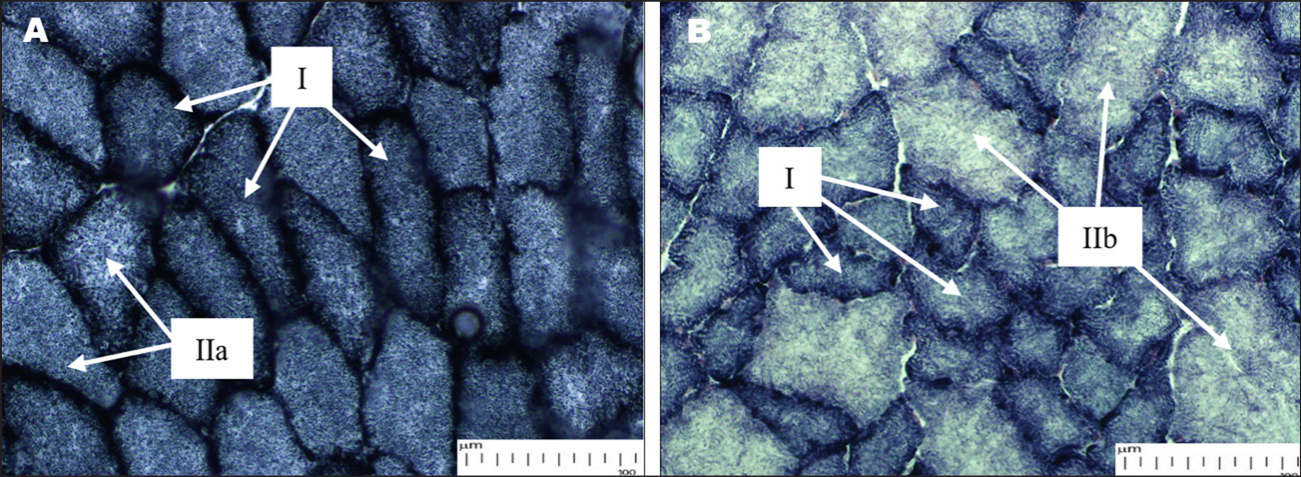
SDH staining of the gastrocnemius muscle from normoglycemic (A) and STZ-induced diabetic rats (B). (A) In normoglycemic rats, SDH activity is intense, indicating characteristics of Type I fibers (*) such as abundant mitochondria and oxidative capacity. Type IIa fibers had intermediate SDH staining, reflecting their mixed oxidative and glycolytic nature. (B) The type I fibers in STZ-induced diabetic rats were not only smaller and less frequent but also showed SDH staining that was surprisingly intense. This marked increase in staining indicates a compensatory mechanism that mitigates mitochondrial dysfunction and maintains oxidative capacity during severe muscle damage. In contrast, the Type IIb (fast glycolytic) fibers were pale in color after SDH staining, suggesting reduced enzyme activity and a greater metabolic insult, which may reflect a shift toward glycolytic metabolism and a decrease in oxidative capacity. These findings highlight widespread mitochondrial defects and remodeling in diabetic muscle.

Two different staining intensities of SDH activity in the gastrocnemius muscle section depict the oxidative capacity of mitochondrial density in muscle fiber (Figure 4). The non-diabetic muscles (Figure 4A) show intense SDH staining, indicating a high mitochondrial content, a characteristic of Type I (slow oxidative) muscle fibers. In comparison, moderate light staining fibers demonstrate intermediate mitochondrial density as well as mixed oxidative and glycolytic metabolism, indicative of Type IIa fibers (fast glycolytic/oxidative). The majority of fibers in STZ-induced diabetic (Figure 4B) are light-stained, suggesting a reduction in oxidative capacity and mitochondrial density. These Type IIb (fast glycolytic) fibers are light and palely stained, indicative of a shift towards glycolysis in diabetes.

### 3.3. Masson Trichrome Staining (MT)

**Figure 5.**
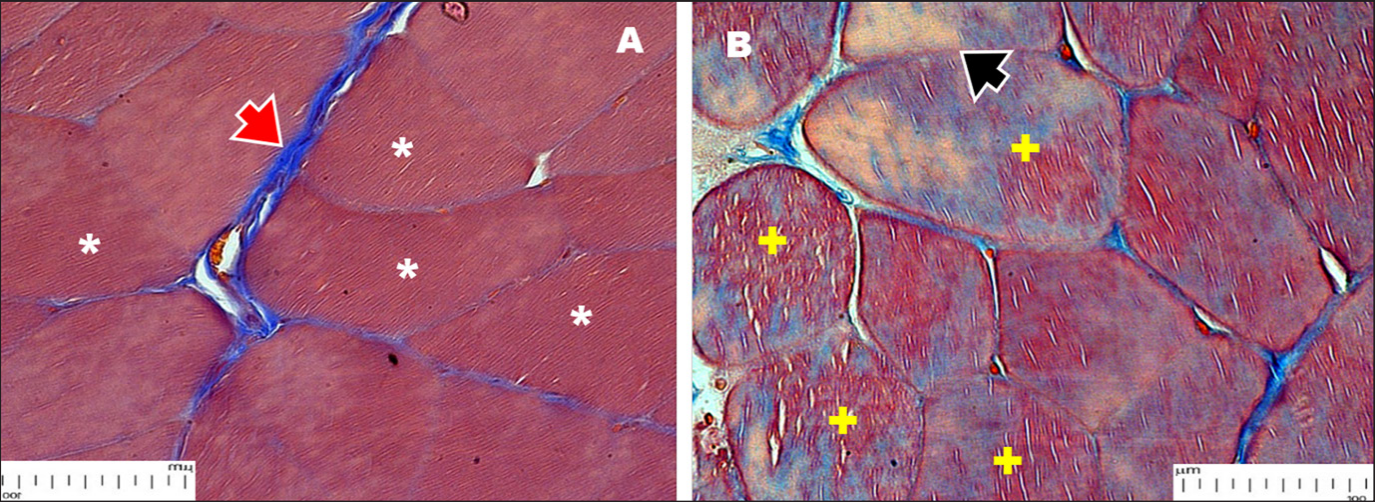
Masson Trichrome staining of the gastrocnemius muscles of normal (A) and STZ-induced diabetic group (B). (A) As observed, in normal rats, a normal pattern of architecture was seen with collagen fibers distinctly visible as blue staining (red arrowhead) in the connective tissue; muscle fibers were red (*). (B) Architectural abnormalities were evident in the STZ-induced diabetic group, with loss of the fiber spaces (black arrowhead). Muscle fibers showed areas of combined blue and red staining (+), indicating collagen deposition.

Masson’s trichrome staining reveals marked structural differences between the muscles of both groups. The muscle samples from normoglycemic rats (Figure 5A) showed a clear distinction between blue collagenous connective tissue and red-stained muscle fibers, indicating a healthy muscle structure. In the STZ-induced diabetic groups, however (Figure 5B), the red and blue staining became intermingled, indicating that collagen has been deposited into intracellular spaces within the muscle fibers. This was in line with significant fiber loss, fibrosis, and disrupted muscle architecture.

### 3.4. Morphological analysis (FESEM & TEM)

**Figure 6.**
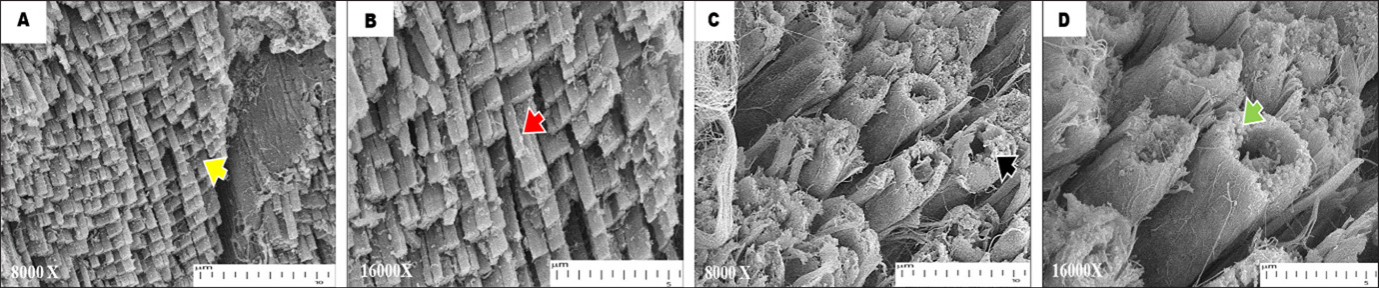
FESEM analysis of the gastrocnemius muscle in the normal (A & B) and the STZ-induced diabetic group (C & D). (A) In the normal group, muscle fibers display orderly alignment with parallel orientation (yellow arrowhead). (B) The sarcoplasm also appeared intact (red arrowhead). (C) In contrast, the STZ-induced diabetic group displayed marked structural degeneration with collapse of the fascicular bundles and loss of myofiber (black arrowhead), forming hollow tubular profiles enclosed by dense fibrous tissue. (D) The outer surface displays a frayed or fibrillar appearance (green arrowhead), representing disrupted endomysial collagen fibrils and remnants of the sarcolemma covering.

FESEM micrograph of the gastrocnemius muscle from the control group (Figures 6A, B) had a preserved ultrastructural profile consistent with normal, healthy skeletal muscle, which was characterized by organized fiber orientation, intact sarcoplasmic organization, and normal morphological integrity.

In contrast, STZ-induced diabetic rats (Figures 6C, D) showed pronounced structural degeneration and fibrosis. The muscle bundle appeared collapsed, with myofibers lost within the fascicle and hollow tubular profiles surrounded by a thick layer of fibrous tissue. The outer surface is “hairy” due to disrupted endomysial collagen fibers and sarcolemma scaffold fragments. These morphological changes in diabetic skeletal muscle are consistent with end-stage fibrotic remodeling and advanced myofiber degeneration.

**Figure 7.**
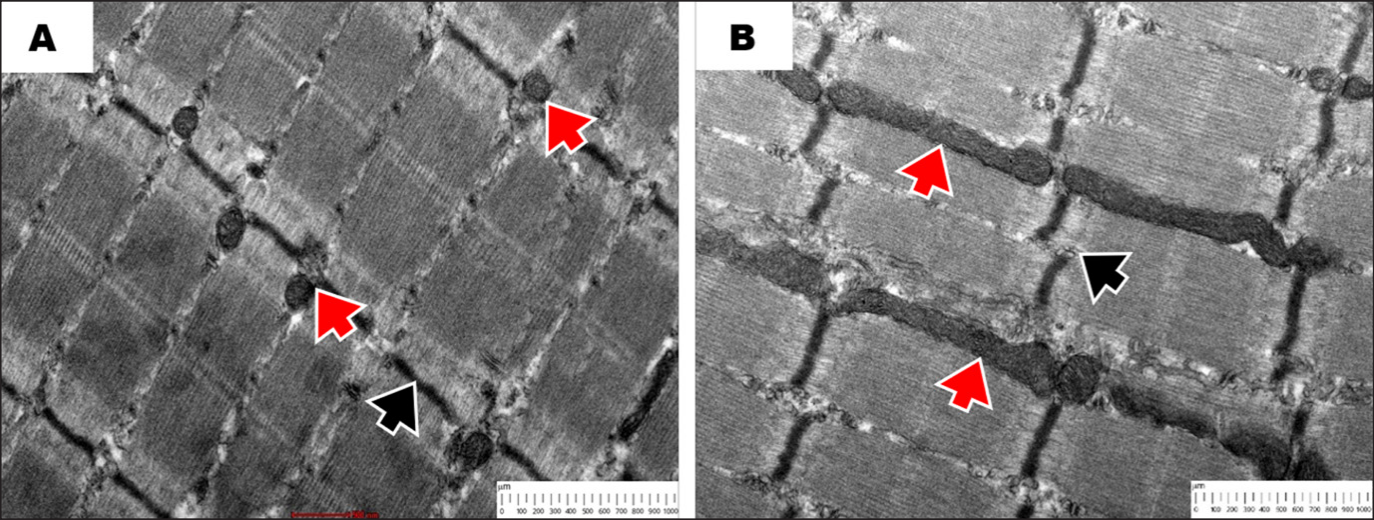
Transmission electron microscopy of gastrocnemius muscles in normal (A) and STZ-induced diabetic groups (B). (A) In normal rats, the ultrastructure of the muscle fibers was well-preserved. The mitochondria were predominantly oval with intact and well-organized cristae (red arrowhead). The Z-discs exhibited regular alignment, reflecting normal sarcomeric architecture (black arrowhead). (B) In contrast, muscles from STZ-induced diabetic rats showed pronounced ultrastructural derangements. Mitochondria appeared elongated and displayed a worm-like, pleomorphic morphology, which is indicative of mitochondrial stress or dysfunction (red arrowhead). The Z-discs were disrupted and misaligned, resulting in a loss of sarcomeric continuity (black arrowhead).

TEM was also used to analyze gastrocnemius samples to evaluate ultrastructural changes in both the normoglycemic and STZ-induced diabetic groups (Figure 7). Our results show that muscle tissue with Z-line alterations and elongated mitochondria can be seen in TEM images of STZ-induced diabetic groups. On the other hand, Z-discs showed regular alignment, and the non-diabetic group had normal muscle morphology, which was characterized by oval mitochondria. These results show significant structural deterioration in the diabetic muscle, which is in line with the pathological remodeling linked to diabetes.

## 4. Discussion

In this study, the T2DM-associated morphological changes in the rat gastrocnemius muscle were investigated using light microscopy and ultra-high-resolution electron microscopy, including FESEM and TEM. We concluded that the diabetic gastrocnemius muscles exhibited significant morphological and metabolic changes, indicative of an early phase of diabetic myopathy development. H&E-stained muscle sections appear to show myofiber necrosis associated with diabetes. There were marked eosinophilic cytoplasmic alterations and a significant loss of material and mass, indicating severe myofiber degeneration and necrosis. Masson’s trichrome staining also demonstrates histopathological changes, including diffuse collagen deposition that replaces native muscle fibers, suggesting progressive fibrotic remodeling. These observations indicate that chronic hyperglycemia can interfere with the organization of skeletal muscle, as seen in myofiber atrophy and fibrosis [17, 18, 19].

Chronic hyperglycemia induces oxidative stress and enhances endoplasmic reticulum (ER) stress in skeletal muscle in diabetes, which activates mitochondrial apoptosis through the induction of the protein kinase RNA-like ER kinase (PERK)–eukaryotic initiation factor 2 alpha (eIF2α)–C/EBP homologous protein (CHOP) pathway [20]. These areas of necrotic fibers and abundant, red-stained eosinophilic cytoplasm are evidence of myofiber degeneration and loss of functional muscle fibers in the H&E section. Mechanistically, this is attributed to increased expression of muscle-specific E3-ubiquitin ligases MuRF1 and atrogin-1 under conditions of chronic hyperglycemia, which subsequently activates the forkhead box O (FoxO) family of transcription factors, leading to an enhanced proteolytic degradation [21]. All these effects contribute to the pathogenic deterioration of diabetic skeletal muscle; specifically, as evidenced by fiber atrophy, reduced thickness, and increased fiber-size heterogeneity, as shown in H&E staining. These results indicate that loss of myofibers and structural abnormalities in diabetic skeletal muscles are linked to a degenerative cascade mediated through oxidative stress and ER stress.

We also found pronounced fibrotic remodeling, with significant collagen deposition replacing native muscle fibers, as evidenced by H&E and Masson’s trichrome staining (Figures 2, 5). During hyperglycemia, signaling cascades, including the transforming growth factor beta (TGF-β) and Notch pathways, are sustainably activated. The Notch signaling pathway was found to regulate cell fate, including differentiation, proliferation, and apoptosis of resident cells [22]. During fibrotic processes, Notch activation promotes the differentiation of resident fibroblasts into myofibroblasts, which are the primary effector cells responsible for the exaggerated production of extracellular matrix (ECM), particularly collagen [23].

Hyperglycemia can induce fibroblast differentiation into myofibroblasts via TGF-β, thereby promoting collagen deposition. These two signaling pathways, as well as Notch, also enhance fibroblast proliferation, ultimately leading to excessive matrix deposition. In fibrotic diseases, TGF-β is upregulated and activated, leading to changes in fibroblast phenotype and function through myofibroblast induction. This leads to the preservation of the extracellular matrix [24]. Research also shows that diabetic patients have higher serum levels of TGF-β1 compared to healthy controls, indicating that TGF-β1 is involved in exacerbating diabetic myopathy [25]. In H&E and Masson’s trichrome-stained sections from diabetic-induced animals, there was architectural collapse, with dense collagenous replacement and fiber atrophy. These processes may result from TGF-β stimulation, as observed in patients with diabetes [25].

Hyperglycemia leads to the formation of advanced glycation end-products (AGEs), which bind to their cell receptors, the so-called receptors for advanced glycation end-products (RAGEs). Persistent activation of this receptor leads to nuclear factor-kappa B (NF-κB)-dependent transcription of pro-inflammatory cytokines and to the recruitment of macrophages [26, 27]. Metabolic dysregulation and metabolic disturbance also favor the amplification of the inflammatory response and recruitment of inflammatory cells [9]. In diabetes, atrogin-1, a muscle-specific F-box protein, was also upregulated and is known to enhance proteolysis in muscle, contributing to muscle wasting [28]. Protein catabolism elevation is also a mediator of the inflammatory process [29]. Macrophages can also indirectly promote fibroblast activation, myofibroblast trans-differentiation, and extracellular matrix deposition by secreting fibrogenic mediators, such as TGF-β [30]. This indirectly results in the formation of collagen cross-links and tissue stiffening, thus facilitating fibrosis remodeling in diabetic skeletal muscles.

Diabetic skeletal muscle stained for COX and SDH showed reduced enzyme activity, suggestive of abnormal mitochondrial oxidation. This decrease is consistent with the effects of chronic hyperglycemia, which lead to the production of reactive oxygen species (ROS) and, consequently, oxidative stress. ROS cause damage to proteins, lipids, and mitochondrial DNA, worsening mitochondrial dysfunction. The reduced COX activity likely reflects defects in mitochondrial biogenesis and function. In combination with diabetes-related myofiber atrophy, these variables result in decreased net mitochondrial content and ultimately a reduction in oxidative capacity [31, 32]. This results in a metabolic adaptation characterized by a conversion of muscle fiber type from Type II to a more glycolytic type in STZ diabetic muscle. Not only will this transition result in diminished mitochondrial enzyme activity, but it will also further impair insulin sensitivity and decrease energetic efficiency in skeletal muscle [19].

Hyperglycemia-induced ROS accumulation also leads to eventual cell death [33]. When ATP consumption increases, and ROS production exceeds the threshold, mitochondria release pro-apoptotic proteins, including cytochrome c, AIF (apoptosis-inducing factor), and Smac/DIABLO, into the cytosol [34]. These proteins activate the caspase cascade, which initiates apoptosis [35]. However, given that anti-apoptotic members of the BCL-2 family protein (e.g., BCL-X_L_) are activated in myofibroblasts, resistance to apoptosis is conferred, and such cells can persist for longer durations despite being primed for apoptotic death [36]. This benefit intensifies extracellular matrix deposition, leading to tissue hardening and, subsequently, fibrosis. Hence, continued myofibroblast activity may be a reason for the more severe fibrosis in long-term diabetic patients, as opposed to the mild fibrotic changes seen in rodent diabetic models [37].

Combined with reduced mitochondrial enzyme activity, fiber type transition, and persistent extracellular matrix deposition, this provides evidence of a coordinated pathological response to long-term hyperglycemia, linking cellular stress to impaired muscle function and fibrotic remodeling. Taken together, the results indicate that long-term hyperglycemia reduces mitochondrial enzyme activity, promotes fiber-type transition, and causes excessive matrix accumulation in muscle, leading to altered contractile properties and fibrotic remodeling.

In the present study, we found that FESEM analysis provided a detailed visualization of the ultrastructural changes in the diabetic gastrocnemius muscle. Endomysial and perimysial fibrosis were observed, as evidenced by irregular muscle fiber appearance, decreased fiber density, and a dense collagenous network between fibers in diabetic muscle. The extracellular matrix of healthy skeletal muscle exhibited highly uniform organization. The endomysium envelops each fiber, while the perimysium contains bundles of muscle fibers, known as fascicles. Since the perimysium is composed of branched collagen filaments, it can maintain fascicle architecture and facilitate the transmission of lateral force [38].

In contrast, FESEM images clearly demonstrate the disarrangement of architecture in diabetic skeletal muscle. Areas of collapse, characterized by myofiber remnants, were one area with a disrupted sarcolemma boundary having a ragged endomysial surface, suggesting the absence of sarcoplasmic and contractile elements. Thus, mechanical coupling between myofibers is lost.

These alterations were mediated by the ubiquitin-proteasome pathway and calpain proteases. These pathways interact to degrade myofibrillar and cytoskeletal proteins, destabilizing the sarcolemma and ultimately leading to myofiber collapse [39]. In addition, podocyte apoptosis mediated by calpain and ubiquitin-dependent proteolysis is enhanced punctually in the tubulointerstitium in diabetic nephropathy. Our report on the modified ultrastructure in diabetic skeletal muscles provides a link between molecular pathways and tissue-level pathology.

The ultrastructural analysis also showed a significantly increased mitochondrial length in STZ-induced diabetic skeletal muscle, implying an imbalance between mitochondrial fusion and fission. Normally, mitochondrial fusion is required for content sharing to help maintain ATP production, while mitochondrial fission enables the removal of damaged organelles by mitophagy [40]. During chronic hyperglycemia, mitochondrial fission, controlled by dynamin-related protein 1 (Drp1), was inhibited by oxidative stress, leading to mitochondrial hyperfusion. This results in the presence of elongated, metabolically impaired mitochondria [41]. Drp1 inhibition also results in decreased mitochondrial translocation of Sdhaf2, which limits the assembly and activity of complex II [41]. Ultimately, mitochondrial respiration and fatty acid anabolism are diminished.

Consistent with these ultrastructural changes, reduced COX and SDH activity observed in this study supports mitochondrial dysfunction and impaired oxidative metabolism in diabetic skeletal muscle. Fragmentation and disorganization of Z-discs in the diabetic gastrocnemius are indicative of extensive sarcomeric derangement and disturbances in mechanical coupling. In this regard, the Z-disc serves as a multifunctional scaffold that tethers actin filaments, linking contractile elements to cytoskeletal and signaling complexes required for mechanotransduction and protein degradation. In addition, the calpain and ubiquitin–proteasome systems, which regulate proteolysis, modulate the activity and stability of structural proteins, including α-actinin, desmin, and telethonin. [42]. Proteolysis leading to progressive Z-disc fragmentation and myofibrillar disruption is likely enhanced by chronic hyperglycemia and oxidative stress, which may be attributed to mitochondrial dysfunction. Mitochondrial localization at Z-discs suggests that defects in bioenergetics also contribute to cytoskeletal dysfunction, linking metabolic stress to mechanical failure. These subcellular alterations are consistent with histological evidence of fiber disorganization and collagen deposition, thereby indicating a transition from sarcomeric damage to interstitial fibrosis.

Beyond this study, although the diagnosis was made by histochemical staining, electron microscopy can serve as a confirmatory tool for diagnosing muscle disorders, especially rare disorders such as congenital myopathy, which are not easily identified by light microscopy. For example, electron microscopy can help determine the subset of the congenital myopathies, such as cylindrical spiral myopathy, zebra body myopathy, fingerprint body myopathy, and intranuclear rod myopathy [43]. Research also found that electron microscopy of skeletal muscle biopsy specimens can help identify and provide definitive diagnoses within three main categories of muscle disorders: Vacuolar, metabolic, and congenital myopathies [43]. For instance, mitochondrial myopathies are characterized by a variety of mitochondrial structures, such as doughnut-shaped mitochondria or onion-shaped cristae, as well as ultrastructural changes, including paracrystalline inclusions [44]. These features are vital not only for diagnosing metabolic diseases, especially mitochondrial diseases, but also for investigating their pathomechanisms. In the present paper, TEM images of mitochondria provided a detailed view of the changes than those from SDH staining. SDH staining only reveals the relative proportions of the population, whereas detailed examination of ultrastructural changes, such as mitochondrial elongation or swelling, is possible via TEM.

Moreover, electron microscopy should also be extended to other muscle degenerative diseases to identify subtle morphological changes in muscle tissue at an early stage that cannot be detected with routine staining. Early degenerative changes, such as sarcolemmal ruptures, disorganization of the extracellular matrix, microcracks in the sarcolemma, or myofibrillar disorganization, may occur at the nanometer scale before any apparent decline in fiber size or number is detectable by light microscopy. Compared with conventional histological techniques, electron microscopy provides earlier and more sensitive evidence of muscle damage by revealing subtle ultrastructural alterations. Although routine clinical workup does not typically use electron microscopy, future research in this area could help monitor the course of muscular dystrophy and possibly contribute to early detection techniques to improve patient outcomes.

## 5. Conclusions

In our investigation, ultra-high-resolution electron microscopy was combined with light microscopy to structurally and ultrastructurally analyze diabetic gastrocnemius muscles. Electron microscopy revealed abnormal mitochondria, sarcomere disarray, and myofibrillar loss, while light microscopy confirmed fiber disarray and connective tissue deposition, linking subcellular changes to whole-tissue remodeling. These results advance the biomechanistic understanding of diabetic myopathy and define ultrastructural signatures with translational implications, establishing the basis for implementing morphological biomarkers in clinical settings and suggesting that aberrant mitochondrial and cytoskeletal configurations may be potential targets for drug intervention. Collectively, our work provides a foundation upon which therapeutic approaches to sustain skeletal muscle structure and function in diabetes may be developed.

## Data Availability

The data presented in this study are available from the corresponding author upon reasonable request.

## List of abbreviations

AGEs, advanced glycation end-products; AIF, apoptosis-inducing factor; ATP, adenosine triphosphate; COX, cytochrome C oxidase; CHOP, C/EBP homologous protein; DAB, 3,3’-diaminobenzidine; Drp1, dynamin-related protein 1; ECM, extracellular matrix; eIF2α, eukaryotic initiation factor 2 alpha; ER, endoplasmic reticulum; FAPs, fibro-adipogenic precursors; FESEM, field-emission scanning microscopy; FoxO, forkhead box O; H&E, hematoxylin & eosin; MT, Masson’s trichrome; NF-κB, nuclear factor-kappa B; NIDDM, non-insulin-dependent diabetic mellitus; OCT, optimum cutting temperature; PERK, protein kinase RNA-like ER kinase; RAGEs, receptors for advanced glycation end-products; ROS, reactive oxidative species; SHD, succinate dehydrogenase; STZ, streptozotocin; T1DM, type 1 diabetes mellitus; T2DM, type 2 diabetes mellitus; TEM, transmission electron microscopy; TGF-β, transforming growth factor beta.
